# In Situ-Forming Microparticles for Controlled Release of Rivastigmine: In Vitro Optimization and In Vivo Evaluation

**DOI:** 10.3390/ph14010066

**Published:** 2021-01-14

**Authors:** Mohamed Haider, Ibrahim Elsayed, Iman S. Ahmed, Ahmed R. Fares

**Affiliations:** 1 Department of Pharmaceutics and Pharmaceutical Technology, College of Pharmacy, University of Sharjah, Sharjah 27272, UAE; iahmed@sharjah.ac.ae; 2 Research Institute for Medical and Health Sciences, University of Sharjah, Sharjah 27272, UAE; 3 Department of Pharmaceutics and Industrial Pharmacy, Faculty of Pharmacy, Cairo University, Cairo 71526, Egypt; ahmed.roshdy@pharma.cu.edu.eg; 4 Department of Pharmaceutical Sciences, College of Pharmacy, Gulf Medical University, Ajman 04184, UAE; dr.ibrahim@gmu.ac.ae

**Keywords:** in situ-forming microparticles, rivastigmine, depot release, sucrose acetate isobutyrate, optimization

## Abstract

In this work, sucrose acetate isobutyrate (SAIB) and polylactic co-glycolic acid (PLGA) were used alone or in combination as a matrix-former (MF) to prepare long-acting injectable rivastigmine (RV) in situ-forming microparticles (ISM). RV-ISM were prepared by the emulsification of an internal phase, containing the drug and the matrix former(s), into an external oily phase containing a stabilizer. The statistical design, Central Composite Design (CCD), was adopted as a quality by design (QbD) approach to optimize the formulation of RV-ISM systems. The fabricated RV-ISM systems was designed to minimize the initial burst drug release and maximize the sustainment of RV release from the ISM and ease of injection. The influence of critical formulation variables such as the matrix-former to drug (MF/D) ratio and SAIB to PLGA (S/P) ratio in the internal phase with respect to critical quality attributes (CQAs), such as the percentage drug release within the first day (Q_1_), the time required for 50% drug release (T_50%_) and the rate of injection, were studied using the CCD. The optimal RV-ISM system with the highest desirability value (0.74) was predicted to have an MF/D ratio of 11.7:1 (*w*/*w*) and an S/P ratio of 1.64:1 (*w*/*w*). The optimal RV-ISM system was assessed for its release profile, injectability, rheological properties, morphology, effect on cell viability, tolerance to γ-sterilization and in vivo performance in male albino rabbits. In vitro release studies revealed that the optimal RV-ISM system released 100% of its drug content throughout a release period of 30 days with only 15.5% drug release within the first day (Q_1_) and T_50%_ of 13.09 days. Moreover, the optimal system showed a high injection rate of 1.012 mL/min, pseudoplastic flow, uniform spherical globules with homogenous particle size, minimal cytotoxicity and high tolerability to γ-sterilization. In vivo pharmacokinetic (PK) studies revealed that the rate of absorption of RV from the optimal RV-ISM system was controlled compared to a drug solution following either intramuscular (IM) or subcutaneous (SC) injection. Furthermore, the optimal RV-ISM was found to follow flip-flop PK with poor correlation between in vitro release and in vivo findings. These findings suggest that the optimal RV-ISM is a promising tool to achieve a sustained release therapy for RV; however, further investigation is still required to optimize the in vivo performance of RV-ISM.

## 1. Introduction

Alzheimer’s disease (AD) is a chronic neurodegenerative disorder characterized by a reduction of cholinergic transmission. AD is considered the most common cause of dementia contributing to about 60–70% of the cases worldwide [1]. Inhibitors of cholinesterase enzymes have emerged as effective agents for the management of AD [2]. Rivastigmine (RV), a phenyl carbamate ester, is a “pseudo-irreversible” dual inhibitor of acetylcholinesterase (AChE) and butyrylcholinesterase (BuChE) that is currently available as oral capsules for the treatment of mild to moderate dementia of the Alzheimer’s type [2,3]. Although RV is rapidly absorbed, its oral bioavailability is limited by first pass metabolism in the intestine and liver mediated by esterase enzymes [4], resulting in inter-patient variation in drug response, ranging from 20% to 60% [4,5]. In addition, the oral administration of RV is associated with dose-dependent adverse effects such as nausea, vomiting and diarrhea which negatively affect the tolerability and compliance of the patient [6].

Many strategies have been attempted to overcome the oral limitations of RV by using drug-loaded liposomes [7], microemulsions [8], nanoparticles [9], transdermal patches [10], buccoadhesive films [11] and in situ-forming oleogel implants [12]. These in situ-forming implants have provided prolonged in vivo drug absorption within the therapeutic range for 11 days with peak plasma levels well below the toxic threshold [12].

In situ-forming systems, such as implants and microparticles, are marked by their ease of administration and less complicated fabrication and demanding manufacturing conditions for sensitive drug molecules [13]. In situ-forming implants were developed as an alternative to solid implants or microparticles formulations [14,15]. These systems are fabricated using hydrophobic polymers, such as poly lactic acid (PLA) and polylactic co-glycolic acid (PLGA), which are dissolved together with the drug in a water-miscible solvent. Upon injection of the drug- polymer solution, the polymer precipitates in body tissues due to solvent/nonsolvent exchange and generates a semisolid or solid depot which can effectively control the release of the drug in vivo [15]. Nevertheless, the use of drug-loaded in situ-forming implants was limited by a typical initial rapid drug release during the formation of the implant, poor rate of injection due to their high viscosity and possible toxicity caused by the use of organic solvents [16].

In situ-forming microparticles (ISM) were developed as an alternative for in situ-forming implants to overcome those limitations [17,18]. The system consists of an internal phase which contains a mixture of the drug and the hydrophobic polymer dissolved in a water-miscible solvent such as N-methyl pyrrolidine (NMP), dimethyl sulfoxide (DMSO) or 2-pyrrolidone. The internal phase is then emulsified into a biocompatible external oily phase such as peanut oil. Following intramuscular (IM) injection, the internal polymer phase precipitates and forms microparticles. ISM systems were reported to have a significantly better rate of injection using high-gauge syringes and lower myotoxicity when compared to the in situ forming implants [16,19,20]. Improved muscle compatibility observed in Sprague Dawley rats and reduced initial burst release of low molecular weight drugs and peptides in vitro and in vivo were related to the lower viscosity of the continuous phase and faster precipitation of the polymer during the formation of ISM [18].

The non-polymeric high viscosity excipient, sucrose acetate isobutyrate (SAIB), is a fully esterified sucrose derivative with a viscosity of over 100,000 cP at room temperature and it is also soluble in organic solvents such as ethanol, dichloromethane ethanol, 2-pyrrolidone and N-methyl-2-pyrrolidone [21,22]. SAIB is biocompatible and biodegradable to inactive metabolites in the body. It has been approved by the US FDA as a food additive and is used as a density-adjusting agent in several non-alcoholic beverages [23]. Studies investigating the use of SAIB as an in situ delivery matrix for drugs and proteins have shown that it is tolerated by animals after subcutaneous (SC) injection in combination with benzyl benzoate, ethanol and benzyl alcohol [21,24]. One characteristic of the SAIB/solvent system is the small amount of organic solvent required during the manufacturing process compared to commonly used injectable PLA- or PLGA-based in situ-forming implants and standard microspheres [21]. Moreover, the resulting SAIB/solvent mixture has a low viscosity, but upon injection, the viscosity increases dramatically as the solvent dissipates away from the injection site. Accordingly, an SC or IM injection of a drug-loaded SAIB/solvent system using simple injection techniques is followed by solvent diffusion into the surrounding tissues that should allow SAIB to form a semi-solid, water-insoluble, biocompatible and biodegradable depot from which the dissolved therapeutic agents are slowly released. Moreover, injectable SAIB implants and microspheres involve shorter and simpler procedures in their preparation and are considered more economical than those prepared using PLGA, which is known to be an expensive polymer [25]. Therefore, drug-loaded ISM systems prepared using SAIB with or without the use PLGA can be tailored to control the release of the drug to achieve optimal plasma level profiles, reduce dosing frequency and minimize the total dose of the therapeutic agent. This should result in fewer side effects, allow more efficient use of the drug and improve patient compliance. Such benefits are particularly valuable in the effective treatment of chronic diseases where patients fail to comply with complex, daily oral medication regimens [21].

The aim of this work is to fabricate and optimize injectable RV-ISM systems, using the principles of QbD, to achieve an extended-release drug depot in the form of microparticles at the injection site as a novel approach to improve the efficacy and tolerance of RV in the treatment of AD. RV-ISM systems were optimized using a series of different ratios of matrix former(s) to drug and SAIB to PLGA according to a central composite statistical design. Then the influence of these formulation variables on critical quality attributes (CQAs) of the prepared RV-ISM systems was investigated. The predicted optimal RV-ISM system showing the optimal in vitro release profile was further characterized for its rheological properties, particle size, morphology, in vitro cytotoxicity and in vivo PK profile following SC and IM administration to rabbits.

## 2. Materials and Methods

### 2.1. Materials

Rivastigmine (RV) was received as a gift from Mepaco Pharmaceutical Co., Elsharqia, Egypt. Poly(d,l-lactide-*co*-glycolide) (PLGA, Resomer^®^ RG 653 H, Mw 24,000–38,000), sucrose acetate isobutyrate (SAIB), sorbitan monostearate (Span 60), sesame oil, N-methyl-2-pyrrolidinone (NMP), Pluronic F68 (PF68), Eagle’s Minimum Essential Medium with Earle’s Balanced Salt Solution (EBSS) and fetal bovine serum (FBS) were purchased from Sigma-Aldrich Co. (St. Louis, MO, USA).

### 2.2. Experimental Design for the Optimization of RV-ISM

A central composite design (CCD) was employed to investigate the influence of several formulation variables on RV-ISM characteristics using Design-Expert^®^ software (Version 12.0, Stat-Ease Inc., Minneapolis, MN, USA). Two numerical factors have been set as the critical material attributes (CMAs) for the experimental design to investigate the effect of these formulation parameters on the selected critical quality attributes (CQAs). These were 1) the matrix former(s) to drug ratio (MF/D *w*/*w*) in the internal phase at three levels (*X*_1_: 4:1, 10:1 and 16:1) and 2) the ratio of non-polymeric SAIB to polymeric PLGA (S/P *w*/*w*) used in the internal phase at three levels (*X*_2_: 0:1, 1:1 and 1:0 *w*/*w*) respectively. The levels of the independent variables were chosen to provide a maximal design space and at the same time enable the feasible processing of the RV-ISM systems (Table 1). On the other hand, three responses have been adopted to be tracked for the optimization of the studied factors: (1) Q_1_, the percentage (%) drug release within the time interval of one day (*Y*_1_); (2) T_50%_, the time elapsed for 50% of the drug to be released from the system (*Y*_2_); and (3) the rate of injection (*Y*_3_) (Table 1). RV-ISM systems were optimized for the responses *Y*_1_–*Y*_3_ to yield the system with the highest overall desirability value. The optimal independent variables were then used to prepare the optimal RV-ISM system.

The statistical design software yielded 11 experimental runs (systems) including 2 replications as presented in Table 2. To increase the predictability of the model and eliminate biased variance, all experimental runs were conducted in random order. All measurements of the three responses were performed in triplicate (n = 3) to satisfy the statistical requirements. All responses were simultaneously fitted to linear, two-factor interaction (2FI), and quadratic response surface models and the resulting polynomial equations were statistically validated by the analysis of variance (ANOVA). Statistical parameters such as *p*-value, adjusted multiple correlation coefficient (Adjusted-*R*^2^), predicted multiple correlation coefficient (Predicted-*R*^2^) and multiple correlation coefficient (*R*^2^) were determined to ensure the significance of the model; 3D response surface plots were generated by Design-Expert^®^ software to analyze the results graphically and determine the degree of interactions between the different factors for each response. The desirability function technique was used to optimize the formulation using numerical and graphical analysis. A desirability value of 0 was considered not acceptable, while a value closer to 1 corresponded to the desired response.

### 2.3. Preparation of RV-ISM Systems

RV-ISM systems were prepared as per the experimental design, through the emulsification of an internal drug-containing matrix-former phase into an external oily phase containing a stabilizer [26]. SAIB and PLGA were used individually or in combination to form the internal phase of the ISM. Briefly, the internal phase was prepared by first dissolving 40 mg of RV in 1 mL NMP containing 2.5% (*w*/*w*) PF68 as surfactant. To this solution, the specified amount of SAIB, PLGA or their physical mixture was added and homogenized using a probe ultrasonicator (Q500, Terra Universal, Inc, Fullerton, CA, USA) until a clear solution was formed. The external oily phase was made of sesame oil containing sorbitan monostearate (2.5% *w*/*w*) as stabilizer. The internal phase and the external oily phase were mixed at a fixed ratio of 1:3 *v*/*v*, respectively, and then emulsified using the Q500 probe ultrasonicator at 50 mHz for 2 min in an ice bath to avoid thermal stresses on the incorporated materials, especially PLGA. The final concentration of RV in each RV-ISM system was 10 mg/mL.

### 2.4. Physico-Chemical Characterization of RV-ISM Systems

#### 2.4.1. In Vitro Drug Release

Aliquots of 1 mL of the prepared RV-ISM systems, equivalent to 10 mg RV, were transferred, using a syringe fitted to a 21-gauge needle, into cellulose dialysis bags (6 cm × 2.2 cm with MWCO of 12–14 kD, Sigma Chemical Company, (St. Louis, MO, USA)) containing 1.5 mL phosphate buffer saline (PBS, pH 7.4) and left for 15 min to form the microparticles [27,28]. An equivalent volume of RV solution in NMP was used as a control to determine the degree of sustainment achieved due to ISM formation. The dialysis bags were then immersed in 20 mL PBS under sink conditions, contained in Stoppard glass bottles, for 30 days in a horizontal water bath shaker (OLS Aqua Pro, Grant instruments, Cambridge, UK) maintained at 37 ± 0.5 °C and operated at an oscillation rate of 100 strokes per minute [29]. At specific time intervals (2 h then 1, 2, 3, 4, 6, 7, 8, 9, 10, 11, 14, 15, 25, 30 days), samples of 1 mL were collected from the receptor medium and were immediately replaced by equal volumes of fresh medium. The samples were then analyzed for drug content spectrophotometrically at λ_max_ 264 nm to calculate the percentage RV released at each time interval. A standard curve of RV in PBS (pH 7.4) was generated over the range of 10–1000 μg/mL and used to convert absorbance to concentration. A cumulative release profile was generated by normalizing the data against the total amount of RV and reported as percentage drug release. All release experiments were conducted in triplicates (n = 3).

The percentage RV released from the prepared RV-ISM systems within the time interval of 1 day (Q_1_) was adopted in the statistical response surface analysis (*Y*_1_) as it reflects the degree of burst drug release. The in vitro release data from all systems were also analyzed according to zero-order, first-order and Higuchi-diffusion release mechanisms [30]. The linear regression equation employed for zero order kinetics was: C_t_ = C_o_ − *k*t; where C_o_ is the zero-time concentration of the drug, C_t_ is the concentration of the drug at time t, and *k* is the apparent release rate constant. First order kinetics were determined according to the equation ln C_t_ = ln C_o_ − *k*t. Drug release following the Higuchi model was determined using the equation Q = *k*t^0.5^, where Q represents the fraction of drug released in time t, and *k* is the Higuchi release rate constant. T_50%_ values (*Y*_2_) were calculated from the slope of the release equation having the highest correlation coefficient (*R*^2^) [31]. T_50%_ was adopted as a statistical response (*Y*_2_) to study the effect of the formulation variables on the rate of RV release from the different RV-ISM systems.

#### 2.4.2. Injectability of RV-ISM Systems

The injectability of the prepared RV-ISM systems was assessed using a handmade equipment depending on controlling the pressure acting on a fixed volume of the studied system and measuring the time required for injection [32]. Samples of 1 mL of the prepared RV-ISM systems were withdrawn into 3 mL syringes fitted to 19-gauge needles. The syringes were then fitted into a rubber tube ending with an air pump to maintain a constant pressure (70 mmHg) on the system surface. The time taken to inject each system was recorded and the rate of injection (mL/min) was determined and considered as an indicator of the ease of injectability [33,34]. All measurements were done in triplicates and the data were reported as mean ± SD.

### 2.5. Rheological Studies

The rheological properties of the optimal RV-ISM system were characterized by generating complete rheograms at room temperature using Cone and Plate Brookfield DV3T Rheometer equipped with Spindle CPE-341 and Rheocalc software (Brookfield Engineering Laboratories Inc., Middleboro, MA, USA) [20]. The shear rate (γ, s^−1^) was plotted as a function of the shearing stress (τ, dyne/cm^2^), and coefficients of viscosity in centipoise (η, cp) were calculated and recorded within a torque range of 10–100%. The shear rate values were kept between 0.3 s^−1^ and 40 s^−1^. The collected data were fitted into the power law model as follows: $\tau =K\;\gamma^{n}$; where *n* (dimensionless) refers to the flow behavior index (*n* = 1 refers to a Newtonian flow, *n* < 1 refers to a shear-thinning Non-Newtonian flow and *n* > 1 refers to a shear-thickening or dilatant Non-Newtonian flow) and *K* is the flow consistency index (dyne/cm s^*n*^).

### 2.6. Particle Size Determination and Morphological Characterization

The average particle size (PS) diameter (z-average) of the optimal RV-ISM system was determined by dynamic light scattering using a Zetasizer Nano ZS (Malvern Instruments, Malvern, UK) and its morphology was examined using transmission electron microscope (TEM). Samples from the optimal RV-ISM system were kept in PBS (pH 7.4) for 15 min at the same conditions used for the in vitro release study to allow complete solidification of the microparticles. Solidified samples were separated, suspended in distilled water and then introduced to the Zetasizer for PS measurement using a clear disposable zeta cell at approximately 25° and an angle of the laser incidence of 173° or dropped on a carbon-coated copper grid for TEM imaging. For TEM studies, the grid was placed in the vacuum chamber of the electron microscope equipped with a LaB_6_-cathode and a Gatan Orius SC1000 camera (Gatan Inc., Pleasanton, CA, USA) at a temperature of −170 °C, using an acceleration voltage of 100 kV (JEOL-2100, JEOL Ltd., Tokyo, Japan) where photomicrographs were captured using different magnifications.

### 2.7. Effect of γ-Sterilization

The optimal RV-ISM system was subjected to γ-sterilization in the presence of dry ice to protect against the heating effect of γ-irradiation [35]. Following the USP recommendations, an effective sterilizing dose of 2.5 Mrad was used. The in vitro drug release profile, injectability rate, rheological properties, and morphology of the optimal RV-ISM system before and after γ-sterilization were compared. The similarity factor (*f*_2_) was calculated to compare the release profiles before (*R*) and after (*T*) sterilization. The following equation was utilized

$$f_{2}=50\times \log\left\{{\left[1+\left(\frac{1}{n}\right)\overset{n}{\underset{j=1}{\sum }}{\left|R_{j}-T_{j}\right|}^{2}\right]}^{-0.5}\times 100\right\}$$

where *j* is the time interval and *n* is the number of sampling intervals considered till both test and reference approach 85% release [36,37]. All measurements were done in triplicate and the data were reported as mean ± SD.

### 2.8. Cell Culture

EBTr (NBL-4) bovine normal tracheal cells (ATCC, Manassas, VA, USA) were cultured in Eagle’s Minimum Essential Medium (EMEM) supplemented with Earle’s Balanced Salt Solution (EBSS), 2 mM Glutamine, 1% non-essential amino acid and 10% heat-inactivated FBS (Sigma-Aldrich Co., St. Louis, MO, USA) under humidified air and 5% CO_2_ at 37 °C. The cells were sub-cultivated 3 times per week at a ratio of 1:4 by trypsinization followed by an addition of fresh growth medium.

### 2.9. Cytotoxicity Studies

The toxicity of the materials used in the fabrication of the optimal RV-ISM system, especially SAIB, was investigated by performing cytotoxicity studies. EBTr (NBL-4) cells were seeded into 96-well plates (Corning, Sigma-Aldrich Co., St. Louis, MO, USA) at a density of 1 × 10^4^ cells per well and left overnight for initial attachment. After 24 h, the cells were treated with fresh media containing a series of concentrations (0.2, 0.4, 0.8 and 1 mg/mL) of drug-free-ISM of the optimal system and left for another 48 h. Cells treated with fresh medium and 1% Triton-X were used as negative and positive controls, respectively. An MTT (3-(4, 5-dimethyldiazol-2-yl)-2, 5-diphenyltetrazolium bromide) cell proliferation and cytotoxicity assay kit (Sigma Aldrich, St. Louis, MO, USA) was used to evaluate the cytotoxicity of the optimal drug-free ISM system by adding 10 μL of 5 mg/mL MTT to each well, followed by incubation at 37 °C for 4 h. The culture media containing MTT were then removed and 100 μL of DMSO were added to each well. The plates were shaken for 20 min followed by measuring the optical intensity at 570 nm using a Synergy™ HTX microplate reader (BioTek, Winooski, VT, USA). Each experiment was performed in triplicate and cell viability was expressed as the percentage of viable cells relative to the positive control using Cell viability (%) = 100 A_s_/A_c_; where A_s_ is the absorbency of the samples and A_c_ is the absorbency of the control.

### 2.10. In Vivo Pharmacokinetic Studies

#### 2.10.1. Study Design

The in vivo studies were carried out to compare the pharmacokinetic (PK) profile of RV from the optimal RV-ISM system to that of a simple RV solution following SC and IM injections of single doses in rabbits. In this study, twelve male albino rabbits (body weight; 3.0–4.0 kg) were randomly assigned to four treatment groups of three rabbits each (n = 3) using a non-blind, four-treatment, randomized parallel design. The four treatments were as follows: Group 1 (SC control) received a SC injection of RV solution; Group 2 (IM control) received an IM injection of RV solution; Group 3 received an SC injection of the optimal RV-ISM system (SC RV-ISM); and Group 4 received an IM injection of the optimal RV-ISM system (IM RV-ISM). The rabbits were supplied by the Laboratory Animal Center at the Faculty of Pharmacy, Cairo University, Egypt and were housed in four cages with free access to water and food and 12 h cycles of day and night. All animal experiments were performed according to ethical principles and the study protocol was approved by the Research Ethics Committee (REC) for Animal Subject Research at the Faculty of Pharmacy, Cairo University, Cairo, Egypt (Approval No. 2355) operating according to the CIOMS and ICLAS international guiding principles for biomedical research involving animals of 2012. Furthermore, all animal experiments comply with directive 2010/63/EU.

#### 2.10.2. Drug Administration and Dosing

IM and SC injections of RV solution were prepared by dissolving RV in NMP to result in a concentration of 25 mg/mL. The optimal RV-ISM system and RV solution were injected in the Hind limb muscles (Gluteal/Quadriceps) of the rabbits, either IM or SC, using a syringe with a 21-gauge needle. The volume of the injection was equivalent to 27.75 mg RV, which represents the monthly dose translated from human to rabbits based on the allometric scaling of the body surface area [38,39].

#### 2.10.3. Blood Sampling

Blood samples (3 mL) were withdrawn from the middle ear vein of each rabbit using syringes with 26-gauge needles at predetermined time intervals post administration of a treatment, into heparinized glass tubes. Blood samples were withdrawn first at 0.5, 1, 2, 3, 5, 7, 10 and 24 h and then each following day until the drug blood concentration could no longer be detected. The plasma was immediately separated from the blood cells by centrifugation at 5000 rpm for 15 min, pipetted into glass tubes and stored frozen at −20 °C until analysis by liquid chromatography-mass spectrometry (LC/MS/MS).

#### 2.10.4. Sample Preparation and LC-MS/MS Analysis

Plasma concentrations of RV following each treatment were determined using a selective, sensitive, and accurate LC-MS/MS method that was developed and validated before use. All chemicals and reagents used were of analytical grade and solvents were of HPLC grade. Before analysis, 20 μL of the internal standard stock solution (100 ng/mL of torsemide) was added to the plasma samples (200 μL) and vortexed. RV and the internal standard was extracted by a liquid/liquid extraction technique through mixing with 500 μL ethyl acetate. The mixtures were vortexed for 3 min and then centrifuged at 5000 rpm. The supernatants were transferred into small glass tubes, evaporated under vacuum (Eppendorf 5301, Hamburg, Germany), then reconstituted with 200 μL of mobile phase. The isocratic mobile phase was a mixture of 80% acetonitrile and 20% 0.02 M ammonium acetate in water. The reconstituted samples were analyzed using liquid chromatography with tandem mass spectrometry LC/MS/MS (Shimadzu, Tokyo, Japan) equipped with a Sunfire column (dimensions: 50 × 4.6 mm, particle size 5 μm; Waters Corp., Milford, MA, USA) and operating at a flow rate of 1 mL/min. All analyses were carried out at room temperature. Multiple reaction-monitoring mode was utilized to detect ions of RV (250.79 Da and 206.30 Da) and torsemide (348.98 Da and 263.90 Da). The output data were processed using Analyst Software V 1.4.2 (AB Sciex Pte. Ltd., Woodlands, Singapore). The utilized LC-MS/MS assay was validated for linearity with *R*^2^ of 0.9916 and a minimum quantification level of 0.1 ng/mL.

#### 2.10.5. Pharmacokinetic Analysis

PK characteristics from plasma data following administration of the four treatments were estimated for each rabbit via non-compartmental PK analysis using Kinetica 2000 software (version 3.0, Kinetica, San Francisco, CA, USA). The observed maximum plasma concentration (*C_max_*, ng/mL) and the time to reach *C_max_* (*T_max_*, h) were estimated directly from the plasma concentration-time profile. The elimination rate constant (*k*, h^−1^) was estimated from the terminal elimination line using the log-linear regression analysis and the half-life (*t*_1/2_, h) was calculated as *t*_1/2_ = 0.693/*k*. The area under the curve, AUC_0–t_ (ng h/mL), was determined as the area under the plasma concentration time curve from time zero up to the last measured time point using the trapezoidal rule. The area under the curve from time zero to infinity, AUC_0–∞_ (ng h/mL) was calculated as AUC_0–∞_ = AUC_0–t_ + C*_t_*/*k* where C*_t_* is the last measured concentration at time *t* and *k* is the terminal elimination rate constant estimated by log-linear regression analysis on data virtually assessed to be a terminal log linear phase. The mean transit time (MTT, h) was calculated from AUMC_0–∞_/AUC_0–∞_ where AUMC_0–∞_ is the area under the first moment curve.

### 2.11. Statistical Analysis

All in vitro experiments were performed in independent triplicates and values are presented as mean ± SD unless otherwise noted. Statistical significance of experiments’ results was assessed by Student *t*-test (two-tailed; *p* < 0.05). For in vivo studies, statistical inferences were based on untransformed values for *C_max_* and AUC variables and observed values for *t*_1/2_. The non-parametric Signed Rank Test (Mann–Whitney’s test) was used to compare *T_max_* between the four treatment groups. The one-way analysis of variance (ANOVA) F-test was used for testing the equality of several means. For multiple comparison, the procedure used was the Least Significant Difference (LSD). Statistical analyses were carried out using SPSS (SPSS^®^ Statistics software program, version 17.0, International Business Machines Corp., Armonk, NY, USA) or Design-Expert^®^ software (Version 12.0, Stat-Ease Inc., Minneapolis, MN, USA).

## 3. Results and Discussion

### 3.1. Design of Experiments and Preparation of RV-ISM Systems

The quality target product profiles (QTPPs) were set up considering the quality characteristics of an injectable RV-ISM system capable of overcoming the limitations of the current RV treatments such as limited oral bioavailability, frequent dosing, adverse effects associated with oral administration, poor patient adherence and serious administration errors. This can be achieved by administering a well-tolerated IM or SC injection of an in situ-forming drug depot capable of controlling the release of RV over an extended period, thus resulting in an optimal systemic therapeutic response and superior clinical outcome for the patient. RV-ISM systems were successfully prepared using the o/o emulsification technique known to yield ISM with small monodisperse particles with relative ease [13]. SAIB, a highly lipophilic water-insoluble sugar and an FDA-approved food additive, was used as a non-polymeric matrix former in the fabrication of RV-ISM systems to test its ability to control the drug release in situ, while making use of its undeniable favorable properties, such as its high solubility in a wide range of organic solvents which result in the formation of low-viscosity solutions compared to more common polymers, in addition to its biocompatibility, biodegradability, in vivo tolerability and low cost [23]. The more expensive and more viscous PLGA is, an FDA-approved biodegradable and biocompatible copolymer, the more widely it is used in the fabrication of drug delivery systems (DDS) that enhance the pharmaceutical characteristics of many drugs [40]. The attractive features of PLGA-based DDS, such as small size, high structural integrity, colloidal stability, ease of fabrication, controlled release capability and surface functionalization, make them very attractive therapeutic delivery vehicles [41,42]. PLGA was used in the fabrication of RV-ISM systems with or without SAIB to study its influence on the CQAs of the prepared systems. NMP was selected as the organic solvent of the internal phase as it is biodegradable, safe to use in parenteral formulations and possesses high solvation power for RV, SAIB and PLGA. The ratio of the internal to the external phase was fixed to 1:3 (*v*/*v*) to form emulsions with a high physical stability and to prevent phase inversion and/or the formation of a flocculated system upon storage while still having the rheological properties that allow them to be easily injected. The nonionic surfactants/stabilizers used in the preparation of RV-ISM systems, such as PF 68 and sorbitan monostearate, are FDA-approved pharmaceutical ingredients known to have good surfactant properties that make their use favorable in many parenteral formulations. The addition of surfactants in the internal/external phase is important to allow easy formation of uniform globules during the emulsification step and prevent aggregation. Entrapment of drugs inside the globules and prevention of drug adhesion to the surface of the globules is also facilitated by the use of surfactants, which can minimize burst drug release.

The response data of the 11 experimental runs (systems), including two replications (F8) using the previously described experimental design, are presented in Table 2.

### 3.2. Determination of Q_1_ and T_50%_ for RV-ISM Systems

The individual effects of the two independent factors (*X*_1_ and *X*_2_) and their interactions (*X*_1×2_) on the three selected responses were statistically analyzed using multiple linear regression analysis and ANOVA was employed to model the data and develop a mathematical expression in the form of a second order polynomial equation, as described below:

$$Y=\beta_{0}+\beta_{1}X_{1}+\beta_{2}X_{2}+\beta_{11}X_{1}^{2}+\beta_{22}X_{2}^{2}+\beta_{12}X_{1}X_{2}$$

where *Y* is the response, *β*_0_ is the intercept coefficient, *β*_1_, *β*_2_, *β*_11_, *β*_22_, *β*_12_ are the linear, quadratic and interaction regression coefficients, and *X*_1_ and *X*_2_ are the studied factors at the specified levels. This equation is used to predict the response for the studied levels of each factor and identify the impact of each factor relative to the other studied factors by comparing the regression coefficients. The predicted *R*^2^ values were in a reasonable agreement with the adjusted *R*^2^ for all responses, indicating that the selected model has predicted all response values with high efficiency (Table 3). The adequate precision, which measures the signal-to-noise ratio, was greater than 4 (the desirable value) for all responses which indicates the ability of the model to describe, navigate and predict data within the design space [43]. 

**Figure 1:**
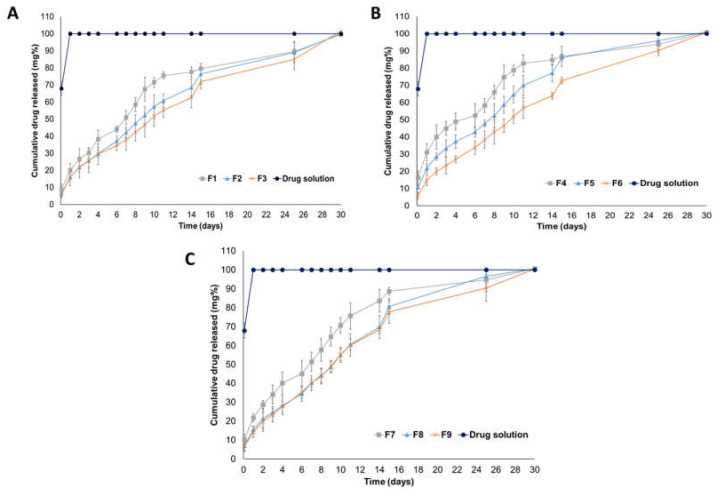
In vitro release profiles of RV from the prepared RV-ISM systems as per the experimental design in phosphate-buffered saline (PBS, pH 7.4) at 37 °C (**A**) F1–F3 systems prepared using PLGA, (**B**) F4–F6 systems prepared using SAIB and (**C**) F7–F9 systems prepared using SAIB/PLGA 1:1, in comparison to RV solution in NMP. Data points are mean ± SD (n = 3 except for F8 n = 9).

Parenteral sustained release systems are designed to act as reservoirs for the incorporated actives to release them over a long period ranging from hours to months with a minimal initial burst drug release. These systems could be modulated to exhibit different drug release patterns and delivery characteristics by varying several formulation variables. All RV-ISM systems demonstrated desirable controlled drug release characteristics in vitro compared to the drug solution, which clearly indicate the capabilities of SAIB, PLGA and their mixture to enclose the drug for an extended period approaching 30 days [44]. The in vitro release profiles of RV over 30 days from the prepared RV-ISM systems as per the experimental design in comparison to RV release from its solution in NMP are shown in Figure 1A–C.

**Figure 2:**
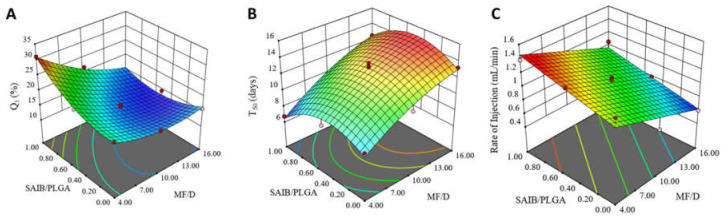
Response surface plots for the effect of MF/D and S/P ratios on: (**A**) Q_1_ (%), (**B**) T_50%_ (days), and (**C**) rate of injection (mL/min).

Two responses were extracted from the in vitro release data: Q_1_ and T_50%_. Q_1_ is considered an indicator for the extent of the initial burst drug release while T_50%_ is used as an indicator for the degree of sustainment of drug release. The graphical analysis of the effects of the studied factors on Q_1_ and T_50%_ of RV-ISM systems are shown in Figure 2A,B.

Q_1_ ranged between 13.3% and 31.1%, as demonstrated in Table 2, which indicates the ability of the prepared RV-ISM systems to control the initial burst release to different extents. These values were in good correlation with previous release findings reported by Vintiloiu et al. regarding the incorporation of dispersed hydrogen tartrate salt of RV into an in situ-forming oleogel implant and were significantly better when compared to release findings obtained from the same study upon incorporation of dissolved RV in the implant [12].

As shown in Figure 2A and Table 3, the two tested formulation variables showed a significant impact on Q_1_ with *p*-values of 0.0004 and 0.0053 for the ratios of MF/D and S/P, respectively. Moreover, the ANOVA test showed a significant two-factor interaction for the effect of MF/D and S/P ratios (*X*_1×2_) on Q_1_ (*p* = 0.0058), indicating the rather complex statistical model for Q_1_. The following quadratic equation was utilized to describe the analyzed data:

*Y*_1_ = 15.32 − 4.65 *X*_1_ + 2.57 *X*_2_ − 3.08 *X*_1×2_ + 2.28 *X*_1_^2^ + 3.09 *X*_2_^2^

According to the statistical analysis, the ability of the prepared RV-ISM systems to control the initial burst release (Q_1_) was significantly improved by increasing the MF/D ratio and decreasing the S/P ratio, as demonstrated in Figure 2A. Increasing the MF/D ratio limited the initial burst release, which might be due to the significant hydrophobicity of both SAIB and PLGA that rendered the matrix more resistant to water permeation and drug diffusion [45,46]. The effect of S/P ratio on Q_1_ was more prominent at the lowest level of MF/D ratio, such that the highest percentage of the initial burst release was observed when the highest S/P ratio, i.e., when the microparticles were fabricated using SAIB alone, was combined with the lowest MF/D ratio which was 4:1. On the other hand, the effect of the MF/D ratio was more significant at the highest level of the S/P ratio. These findings indicate that SAIB, as a non-polymeric matrix former, was less effective in reducing the initial burst release from the prepared RV-ISM systems when compared to polymeric PLGA. This could be due to the formation of a less viscous internal phase when SAIB is used alone, which during emulsification into the external oily phase may allow more rapid drug diffusion compared to PLGA, leading to drug dissipation to the outer phase and/or distribution near the surface of the internal phase droplets. Lower viscosity of the internal organic phase is also expected to decrease the magnitude of SAIB-SAIB and SAIB-oil hydrophobic interactions, which might result in the formation of smaller droplets and redistribution of the drug at the internal/external interface, leading to a higher initial burst release during the sol/gel (solidification) transformation process.

The kinetics of drug release from the prepared RV-ISM systems were also subjected to statistical analysis according to different models. The release kinetics were found to best fit the Higuchi model except for F1 and F2 systems, which followed a zero-order kinetic pattern, further indicating that the studied formulation variables were critical and had a significant effect on the release kinetics.

The ability of the different RV-ISM systems to sustain the drug release for a long period was assessed by calculating the T_50%_ values from their release profiles. The average T_50%_ values ranged from 7 to 15 days, as reported in Table 2. The polynomial equation describing the correlation between the tracked independent variables and T_50%_ was as follows:

*Y*_2_ = 12.95 + 2.47 *X*_1_ − 0.53 *X*_2_ + 0.27 *X*_1×2_ − 0.8 *X*_1_^2^ − 2.15 *X*_2_^2^

As presented in Table 3 and graphically illustrated in Figure 2B, the MF/D ratio was the only factor having a significant effect on the T_50%_ response (*p*-value = 0.0002). The statistical analysis showed that using a higher MF/D ratio was associated with significantly longer T_50%_ values, which is consistent with the Q_1_ results in that higher amounts of matrix former(s) render the matrix more resistant to water permeation, resulting in slower drug diffusion. These results also suggest that SAIB, PLGA or their mixture have a similar sustainment effect once the microparticles are formed. Similar results were observed by Vintiloiu et al., who developed in situ oleogel implant containing RV [12].

### 3.3. Determination of the Rate of Injection

Syringeability, which is the force required to discharge the injected system through the syringe needle, is a critical quality attribute for any injectable pharmaceutical product for ease of administration. The matrix former type as well as its inherent viscosity, its concentration as well as the type of oil used in the formation of ISM systems have been reported to play a significant role in the syringeability of the final product [19].

The average rate of injection of the prepared RV-ISM systems ranged from 0.6 and 1.38 mL/min as demonstrated in Table 2. As shown in Figure 2C and Table 3, the two tested formulation variables showed a significant impact on the rate of injection with *p*-values of 0.0003 and 0.0028 for the ratios of MF/D and S/P, respectively. The rate of injection equation obtained from the analysis was

*Y*_3_ = 1.04 − 0.25 *X*_1_ + 0.17 *X*_2_

According to the statistical analysis, a decreasing MF/D ratio and an increasing S/P ratio significantly enhanced the injectability of the system. The inverse relation between MF/D ratio and the rate of injection could be attributed to the overall increase in the viscosity of the system as the amount of the incorporated matrix former is increased [47]. On the other hand, SAIB was found to have a higher rate of injection when compared to PLGA, which might be due to the relatively lower viscosity of SAIB solution compared to PLGA as previously discussed and reported [22]. These results were also consistent with the results obtained for Q_1_ and T_50%_ responses.

### 3.4. Selection of the Optimal RV-ISM System

**Figure 3:**
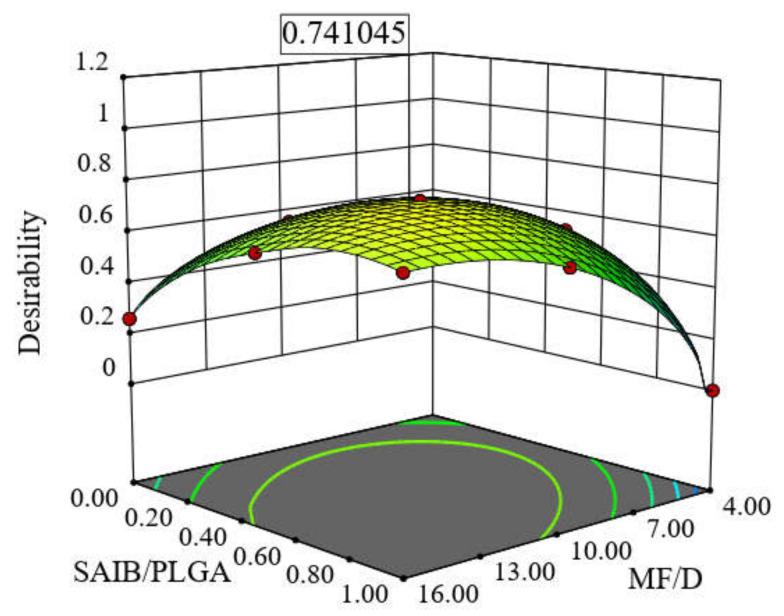
Optimization of RV-ISM systems showing the response surface plot for the effect of using MF/D in the ratio 11.71:1 and S/P in the ratio 1.64:1 on the desirability value.

The response surface analysis of the CCD was used to predict the optimum levels of the studied factors for the preparation of the optimal RV-ISM system. The RV-ISM system was optimized to have the lowest Q_1_, the longest T_50%_ and the fastest rate of injection. The highest desirability value (0.741) for the simultaneous optimization of all responses is shown in Figure 3, which was depicted in the system prepared using 11.71:1 (*w*/*w*) MF/D ratio and 1.64:1 (*w*/*w*) S/P ratio (62.12% SAIB). The optimal RV-ISM system selected by the statistical design was prepared and characterized for its release profile and rate of injection. The optimal system released 100% of its drug content throughout a release period of 30 days, released 15.51% of the drug within the 1st day (Q_1_), showed an observed T_50%_ of 13.09 days and had an observed rate of injection of 1.012 mL/min. These observed results deviated from the predicted values by 5.3%, 2.56% and 0.29% for Q_1_, T_50%_ and rate of injection, respectively as shown in Table 4.

Based on these results, it can be concluded that the optimal RV-ISM system, guided by QbD, provides a promising formulation for the easy fabrication of a readily injectable ISM system that can control the drug release with a minimal initial burst release; therefore, it was selected for further investigation.

### 3.5. Rheological Properties of the Optimal RV-ISM System

**Figure 4:**
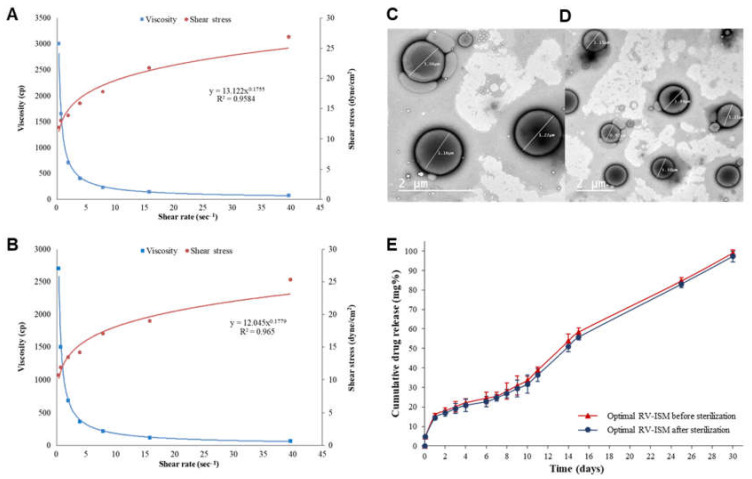
Effect of **γ**-sterilization on the rheological properties of the optimal RV-ISM system: (**A**) Before sterilization and (**B**) After sterilization; TEM of optimal RV-ISM system: (**C**) Before sterilization; and (**D**) After sterilization; and (**E**) In vitro release profiles of the optimal RV-ISM system in PBS (pH 7.4) at 37 °C before and after sterilization. Data points are mean ± SD (n = 3).

The rheological characteristics of the optimal RV-ISM system are of great importance since they can affect viscoelasticity, spreadability, injectability, bioadhesion, tolerability, in vitro release, in vivo release as well as in vivo PK. The rheological studies demonstrated that the optimal RV-ISM system exhibited Non-Newtonian behavior characterized by shear-thinning as shown by a drop in viscosity at an increasing rate of shear with a flow index of 0.1755 (Figure 4A). The system exhibited a mainly pseudoplastic flow typical of polymeric dispersions, with its curve beginning very close to the origin at low rates of shear. The observed shear-thinning behavior allows for faster administration and might enable the use of high-gauge needles, and hence reduce patient discomfort.

### 3.6. Particle size Determination and Morphological Characterization

The PS is an important parameter that can affect the drug release, injectability and stability of RV-ISM systems. The solidified optimal RV-ISM system was examined using dynamic light scattering and TEM and characterized for particle size, shape, surface morphology and homogeneity. The optimal RV-ISM system showed an average PS of 1.3 ± 0.1 μm. TEM micrographs showed spherical, smooth and uniformly shaped microparticles as illustrated in Figure 4C. The microparticles appeared to be well dispersed with minimal aggregation and their size was within the range of 1 μm to 1.5 μm. The PS determined by TEM was in a good alignment with the PS measurements obtained through the Zetasizer. This might be due to the surface-active characteristics of PF68 and Span 60 used in the internal and external phases that facilitated the formation and stabilization of the microparticles. The formation of a well-dispersed non-flocculated system is extremely important in injectables as particle aggregation may alter the ease of injection, tolerability, drug release characteristics and possibly the PK profile of the drug.

### 3.7. Effect of γ-Sterilization

The effects of γ-sterilization on the in vitro drug release, rate of injection, rheology and morphology of the optimal RV-ISM system were investigated. The drug release profiles before and after γ-sterilization were compared by a model-independent approach using the similarity factor (*f*_2_). Results showed that the mean release values from both profiles at each time interval are not statistically significantly different, with an *f*_2_ value of 73 ensuring the equivalence of the two profiles (Figure 4E). It is worth noting that the in vitro drug release profile from the optimal RV-ISM system showed a characteristic triphasic pattern. The first phase was characterized by an initial burst release with about 15% of the drug being released in the first 24 h, a second phase where drug release might be controlled by its diffusion through the ISM matrix and finally a third phase starting from day 12 characterized by an increase in the rate of drug release probably due to matrix degradation and erosion over time [48]. The flow rate of the sterilized RV-ISM system through a 19-gauge needle under a pressure of 70 mmHg remained at the level 1 mL/min, indicating that the sterilization process did not alter the injectability of the system. Furthermore, no change in the rheological characteristics of the sterilized RV-ISM system were observed, as the optimal system retained its pseudoplastic flow with a flow index of 0.1779, as shown in Figure 4B. Similar morphological characteristics were observed upon TEM imaging of the sterilized system, including the size, regular spherical outline, smooth surface and absence of aggregation (Figure 4D). All these findings confirmed the suitability of γ-sterilization for maintaining the morphological, physical and pharmaceutical characteristics of the developed optimal RV-ISM system.

### 3.8. In Vitro Cytotoxicity Studies

In this study, the optimal RV-ISM system was fabricated using 62.12% SAIB and 37.88% PLGA. PLGA, an FDA-approved copolymer, is widely used in drug delivery and is known by its good biocompatibility and biodegradability. On the other hand, SAIB is used as a food additive and has been approved by US FDA at a daily intake of up to 20 mg/kg of body weight [49]. However, the use of SAIB in a drug delivery system, such as the one developed in this work, would require its biocompatibility and low toxicity with the surrounding tissues at the local injection site. The cytotoxic potential of different concentrations of the optimal drug-free ISM system on normal cell viability and proliferation, using an EBTr (NBL-4) cell line, relative to cells treated with fresh medium and 1% Triton-X, as negative and positive controls respectively, was assessed by performing an MTT assay.

**Figure 5:**
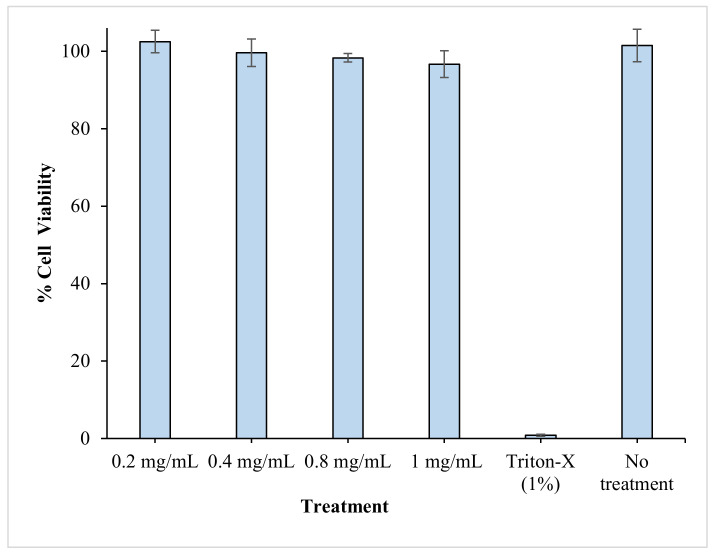
In vitro cytotoxicity results using EBTr cells treated for 48 h with different concentrations of the optimal drug-free-ISM system. Data points are mean ± SD (n = 3).

MTT assay indirectly reflects the number of viable cells and is often used to determine the cytotoxic effects of toxic substances on cells. As shown in Figure 5, treating the cells with a drug-free optimal ISM system for 48 h did not result in any significant decrease in % cell viability relative to the positive control, even when the cells were treated with a high concentration of drug-free ISM systems (1 mg/mL), indicating the biocompatibility and low cytotoxicity of the developed microparticles.

### 3.9. In Vivo Pharmacokinetic Studies

**Figure 6:**
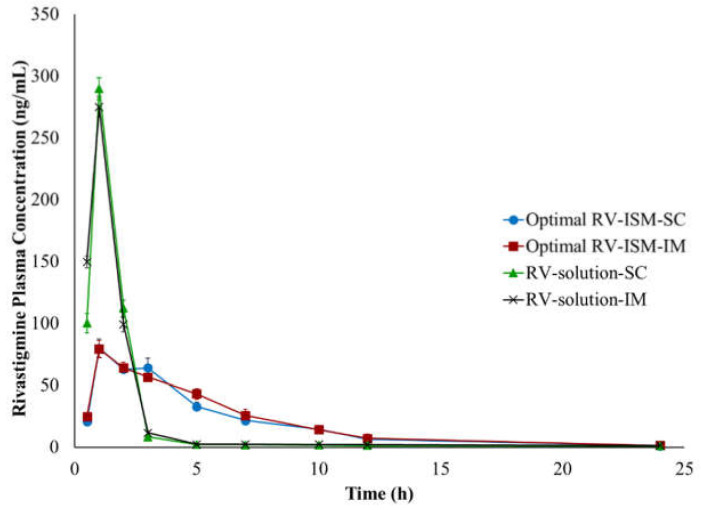
Mean (±SD) plasma RV concentrations following IM and SC injections of the optimal RV-ISM system and RV solution to albino rabbits. Data points are mean ± SD (n = 3).

The in vivo characteristics of the optimal RV-ISM system were assessed and compared to those obtained from RV solution by monitoring RV plasma levels following IM and SC injection to rabbits using a parallel design. The mean plasma concentration time curves obtained from the optimized RV-ISM system and RV solution are demonstrated in Figure 6, while the mean PK parameters obtained from the four treatments are summarized in Table 5. A remarkable difference in the rate of drug absorption from the optimal RV-ISM system and RV solution was observed.

The plasma profiles of the RV solution resulting from the IM and SC injection showed significantly (*p* < 0.05) higher plasma concentrations up to 2 h post administration compared to the optimal RV-ISM system followed by fast elimination and rapid decline in RV concentrations in the subsequent time intervals. The IM injection of the RV solution showed significantly higher RV plasma concentrations after 30 min compared to the SC injection, which is expected, as the blood perfusion in the SC tissue is lower than in the muscles resulting in a slower drug absorption with respect to the IM injection [50]. On the other hand, the optimal RV-ISM system exhibited a sustained in vivo profile characterized by a significantly lower *C_max_* and a slowly declining curve that maintained RV plasma concentration for a prolonged time. The initial rapid increase in RV concentration up to 1 h could be due to burst drug release.

Results showed there was no difference in RV plasma concentrations at all time points between the IM and SC injection of RV-ISM, indicating the rapid formation of the microspheres upon injection regardless of the injection site, which is important to minimize the initial burst release during the sol/gel transformation process in vivo. SC injections are the most often used parenteral administration route due to the possibility of self-injection by the patients, especially if the administration volume is small. The findings of the in vivo study also showed that the bioavailability of the optimal RV-ISM system relative to the RV solution was about 20% and 12% higher from the IM and SC injection, respectively. This is an indication of the absence of any decline in RV bioavailability from the developed sustained release formulation.

It was noticed that the half-life of RV calculated from the plasma concentration data of the optimal RV-ISM system was statistically significantly higher (3.82 h) compared to the half-life calculated from the plasma concentration data of the RV solution (0.54 h). This finding is not consistent with the pharmacokinetic theory in which drug absorption should not alter the elimination of the drug [51]. This suggests that the optimal RV-ISM system follows a flip-flop PK, with absorption being much slower than elimination of the drug from the blood. This causes the absorption rate constant (k_a_) to be the rate limiting step (k_el_ > k_a_), making it slower and causing an increase in half-life [52]. These results also indicate that the diffusion and release of RV from the oily RV-ISM depot at the injection site is the rate-limiting step due to slower k_a_ than k_el_. Flip-flop PK are widely reported for drugs administered in modified-release dosage forms. For example, when a thermoreversible poloxamer gel incorporating paclitaxel in liposomes was injected subcutaneously to mice, it showed an apparent half-life of 15 h compared to a half-life of 2.4 h from the gold standard Cremophor El paclitaxel intravenous injection, which was attributed to flip-flop PK [53]. In another study, a long-acting naltrexone extended-release formulation, based on microspheres incorporated into a biodegradable polymer matrix of polylactide-coglycolide, was developed to have continuous exposure for 1 month for the treatment of alcohol dependence following IM injection. The product was found to have a long apparent half-life (5–8 days) for naltrexone, which was attributed to the slow release of naltrexone and k_a_-limited elimination or flip-flop PK [54].

Flip-flop PK was further confirmed by the significantly higher mean MTT estimate calculated from the optimal RV-ISM system (5.73 h) compared to RV solution (1.33 h), which might be due to the significantly higher mean absorption time (MAT) taken by the drug molecules to be absorbed into the systemic circulation from the optimal RV-ISM system compared to the RV solution.

Although the optimal RV-ISM system was able to sustain the in vivo release and the PK profile of RV, however, the achieved sustainment was not matching the in vitro extended release profile, which lasted for a period of one month. Several scenarios might be responsible for the observed lack of correlation between the in vivo and in vitro results. One such scenario could be due to the susceptibility of SAIB to be rapidly metabolized after injection by a non-specific esterase enzyme available in high concentration in blood and/or tissue phagocytes [55,56]. SAIB was previously utilized to develop in situ-forming implants of other drugs, such as ropivacaine, lornoxicam and risperidone; it was capable of yielding a depot PK profile [21,26,44,57]. The faster in vivo release observed in this study might also arise from the relatively small size of the injected microparticles from the optimal RV-ISM system (ranging from 1 μm to 1.5 μm) compared to the much larger marketed microparticles (ranging from 25 μm to 180 μm), which was possibly optimum for opsonization followed by rapid phagocytosis and SAIB lysis by a non-specific esterase enzyme into soluble sugar esters [58]. Alternatively, the lack of correlation could be attributed to the observed shear-thinning rheological behavior of the system, which might lose viscosity in vivo following injection due to the strong movement of the muscles in rabbits. Consequently, this might increase the possibility of dose dumping after the IM injection [59,60].

Another explanation may arise from spontaneous external and internal morphological changes of the microparticles during the initial period of burst release. It has been reported that the in vivo performance of injectable long-acting formulations is extremely challenging to predict based on in vitro release studies and that is why only about 20 injectable long-acting depot products reached the market in the last 30 years [61].

These results suggest that further studies are needed to optimize the in vivo performance of RV-ISM. Future investigations will focus on studying the effect of the microparticle size and viscosity of the system on the in vitro-in vivo correlation. However, the optimal RV-ISM system developed in this work could be a replacement or alternative for the daily RV transdermal patch. Alternatively, the developed ISM system could be used as a delivery platform that can be suited for various injectable therapeutic applications through IM or SC administration.

## 4. Conclusions

Injectable RV-ISM systems were successfully prepared using SAIB and PLGA with a sustained drug release pattern and a high rate of injection. A central composite design was utilized as a QbD approach to optimize the formulation of RV-ISM systems. The optimal RV-ISM system was characterized by a pseudoplastic rheological behavior, homogenous spherical non-flocculated microparticles, good stability after δ-sterilization and low cytotoxicity. PK studies revealed that the optimal RV-ISM system showed a sustained in vivo blood profile characterized by flip-flop PK when compared to an RV solution after IM and SC injection. The observed in vivo sustainment did not match the in vitro release profile in extending the drug release for a period of one month. These findings suggest that the optimal RV-ISM system developed in this work was not capable to achieve the desired in vivo depot release in its current form, but instead can be used to achieve a sustained release therapy for a maximum of one day, which might be suitable for elderly patients with difficulty in swallowing to replace oral administration or daily transdermal patch. We also emphasize in this study the uncoupling of the in vitro release characteristics with the in vivo release characteristics, which impose a big challenge in the development of injectable controlled-release systems.

## Figures and Tables

**Table 1:** Independent variables (factors) and dependent variables (responses) for the optimization of RV-ISM systems.

Numerical Factors		Applied Levels		
		Low (−1)	Medium (0)	High (+1)
*X*_1_	MF/D (*w*/*w*)	4:1	10:1	16:1
*X*_2_	S/P (*w*/*w*)	0:1	1:1	1:0
**Responses**		**Optimization Goal**		
*Y*_1_	Q_1_ (%)	Minimize		
*Y*_2_	T_50%_ (days)	Maximize		
*Y*_3_	Rate of injection (mL/min)	Maximize		

**Table 2:** Experimental design and measured responses for the optimization of RV-ISM systems.

Formulation	MF/D (*X*_1_)	S/P (*X*_2_)	*Y*_1_: Q_1_ (%)	*Y*_2_: T_50%_ (Days)	*Y*_3_: Injection Rate (mL/Min)
F1	4:1	0:1	20.1 ± 1.5	8.54 ± 0.3	1.24 ± 0.09
F2	10:1	0:1	16.0 ± 1.3	10.59 ± 0.7	0.60 ± 0.03
F3	16:1	0:1	16.0 ± 2.1	13.24 ± 1.1	0.60 ± 0.04
F4	4:1	1:0	31.1 ± 1.7	6.74 ± 0.4	1.38 ± 0.11
F5	10:1	1:0	21.7 ± 0.9	9.96 ± 0.1	1.11 ± 0.08
F6	16:1	1:0	14.7 ± 1.6	12.51 ± 1.4	1.06 ± 0.05
F7	4:1	1:1	21.8 ± 0.8	8.64 ± 0.6	1.30 ± 0.02
*F8	10:1	1:1	16.3 ± 1.4	13.10 ± 1.3	1.08 ± 0.01
			15.5 ± 1.1	13.61 ± 0.7	1.03 ± 0.10
			13.3 ± 1.0	13.20 ± 1.2	1.11 ± 0.03
F9	16:1	1:1	14.3 ± 1.5	14.61 ± 1.0	0.80 ± 0.06

Data are mean value ± SD (n = 3), *F8 system was prepared in triplicate.

**Table 3:** Output results of the experimental design.

Response	Model Equation (*p*-Value)	Lack of Fit (*p*-Value)	Adjusted *R*^2^	Predicted *R*^2^	Adequate Precision	Significant Terms
Q_1_ (%)	Quadratic (*p* = 0.011)	0.6951	0.9333	0.8236	18.3	*X*_1_ (*p* = 0.0004) *X*_2_ (*p* = 0.0053) *X*_1×2_ (*p* = 0.0058)
T_50%_ (days)	Quadratic (*p* = 0.0011)	0.0978	0.9334	0.7685	17.19	*X*_1_ (*p* = 0.0002)
Rate of injection (mL/min)	Linear (*p* = 0.0003)	0.1305	0.8421	0.7177	16.19	*X*_1_ (*p* = 0.0003) *X*_2_ (*p* = 0.0028)

**Table 4:** Optimized parameters along with predicted and observed values of responses.

Variables	Values	Responses	Predicted Values	Observed Values
*X*_1_	11.71:1 (*w*/*w*)	*Y*_1_ (Q1)	14.774%	15.518%
*X*_2_	1.64:1 (*w*/*w*)	*Y*_2_ (T_50%_)	13.436 days	13.09 days
		*Y*_3_ (Rate of injection)	1.009 mL/min	1.012 mL/min

**Table 5:** Mean PK parameters of RV following SC and IM injection of the optimal RV-ISM system or RV solution to rabbits.

PK Parameter	Optimal RV-ISM System		RV Solution	
	SC Injection	IM Injection	SC Injection	IM Injection
*C_max_* (ng/mL)	79.95 ± 7.26	79.25 ± 6.35	275.00 ± 4.26	289.77 ± 7.59
*T_max_* (h) *	1.00	1.00	1.00	1.00
AUC_0–24_ (ng·h/mL)	440.39 ± 12.70	465.69 ± 10.54	400.03 ± 6.21	394.65 ± 9.37
AUC_0–∞_ (ng·h/mL)	447.50 ± 9.33	473.32 ± 8.02	402.01 ± 8.95	396.37 ± 10.48
AUMC_0–∞_ (ng·h/mL)	2509.76 ± 25.66	2714.36 ± 32.33	517.70 ± 11.89	526.08 ± 3.94
k_el_ (h^−1^)	0.18 ± 0.01	0.18 ± 0.08	1.22 ± 0.05	1.27 ± 0.04
t_½_ (h)	3.78 ± 0.28	3.82 ± 0.32	0.57 ± 0.03	0.54 ± 0.05
MTT (h)	5.61 ± 0.47	5.73 ± 0.16	1.29 ± 0.10	1.33 ± 0.14

Data are the mean values (n = 3) ± SD; * Data are the median value.

## Data Availability

The data presented in this study are available within the article or on request from the corresponding author.
